# Smart Biomaterials: An Evolving Paradigm in Dentistry

**DOI:** 10.7759/cureus.47265

**Published:** 2023-10-18

**Authors:** Harsha P Rathi, Manoj Chandak, Amit Reche, Abhilasha Dass, Swayangprabha Sarangi, Samiksha R Thawri

**Affiliations:** 1 Public Health Dentistry, Sharad Pawar Dental College and Hospital, Datta Meghe Institute of Higher Education and Research (Deemed to be University), Wardha, IND; 2 Conservative Dentistry and Endodontics, Sharad Pawar Dental College and Hospital, Datta Meghe Institute of Higher Education and Research (Deemed to be University), Wardha, IND

**Keywords:** smart ceramic, smart composite, smart behavior, biosmart, smart materials

## Abstract

According to definition and general agreement, smart materials have properties that can be altered in a controlled fashion by stimuli including stress, temperature, moisture, pH, and electric or magnetic fields. Various recent materials in materials science are in working order, meaning they must achieve their tasks and should go through intentional modification. Smart materials change one or more of their characteristics in response to inputs. They can be called as responsive materials. As these materials have been available for so long, they are used for a wide range of purposes. These qualities have useful applications in many different industries, including dentistry. Zirconia, shape-memory alloys, and SmartSeal obturation system (Prosmart-DRFP Ltd., Stamford, United Kingdom) are a few examples of dental materials with intelligent behavior. The creation of novel materials is a major trend in materials science. These materials might make it possible to develop cutting-edge dental therapies with vastly improved clinical results. This article reviews the following: nickel-titanium smart alloy, smart composites, self-healing composites, smart ceramics, glass ionomer cement as a smart material, SmartSeal obturation system, and smart coatings for dental implants. We can better understand these biosmart materials with the aid of this review. The development of these newer and superior smart materials makes the outcome of the treatment far better for both the operator and the patient.

## Introduction and background

Materials science has changed. Earlier dental materials were passive and inert, meaning they interacted with human fluids and tissues sparingly or not at all [1]. Situation in hand is altered; the smart biomaterials react in the presence of saliva and other environmental factors. Various recent materials in materials science are in working order, meaning they must achieve their tasks and should go through intentional modification. Smart materials change one or more of their characteristics in response to inputs. They actively contribute to the functionality of the structure or apparatus. The knowledge about liberation of fluoride from the various dental cements, restorative materials, etc. may have been an indication that an active instead of a passive substance can be appealing in dentistry [2].

On the basis of how they interact with the environment, materials used in dentistry are classified as bioinert (passive), bioactive, and bioresponsive or smart materials [3]. The definition of smart material is "the designed materials that have one or more properties that can be significantly changed in a controlled fashion by external stimuli, such as stress, temperature, moisture, pH, and electric or magnetic fields" [4]. Smart materials can be called as responsive materials. As these materials have been available for many years now, they are used for a wide range of purposes. When a substance can detect environmental stimuli and respond to it in a practical, trustworthy, and typically reversible way, it is said to exhibit smart behavior. An extremely effective material can use the response to the stimuli from the outside world to start or activate an active response [5].

Smart materials may develop accidentally or may be intentionally built with intelligence [6]. According to some scientists, no material can truly exhibit smart behavior and these are merely responsive materials. They maintain that intelligence comprises concepts like adaptation and feedback in addition to just making a reaction in relation to a stimulus. Others distinguish between being simply smart and being actually intelligent, in the sense of having the capacity to carry out tasks like making decisions or taking care of oneself. In this sense, no artificial substance exists yet.

Smart biomaterials are such advances made in dentistry which led to the formation of materials that change themselves when any kind of environmental factors such as heat, light, moisture, pH, etc. is implemented on them. This leads to the formation of a material that has even more superior properties than the conventional materials. As a result of the development of these newer and superior smart biomaterials, the outcome of the treatment gets improved for both the operator and the patient. This shows a shift in material philosophy from conventional to smart biomaterials. As the world is advancing in many new technologies, there is a need to implement smart biomaterials. This review will help to understand biosmart materials better. This article seeks to provide a description of various dental materials that behave smartly.

## Review

Nickel-titanium (NiTi) smart alloy

Terms such as smart material and smart behavior are given in relation to NiTi alloys commonly known as shape-memory alloys (SMAs). In endodontics, 55 wt.% nickel and 45 wt.% titanium, also known as "55NiTiNOL," are frequently employed [7]. In 1988, Walia et al. introduced NiTi to the field of endodontics. Two prominent characteristics of NiTi alloys, "superelasticity" and "shape memory," are what give them their smart behavior. The ability of the substance to go through a phase transition (this is sort of atomic ballet in which the solid partials gently rearrange themselves in reaction to stimulation such as fluctuations in the temperature or applying mechanical stress) is what gives rise to this "smart" attribute.

**Figure 1:**
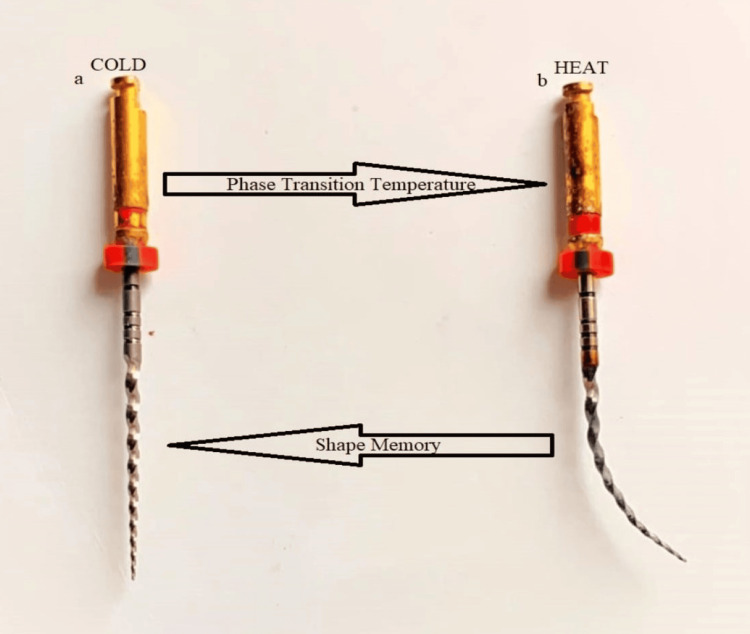
Phase transition and shape memory of a NiTi file NiTi: nickel-titanium Image Credit: Harsha P. Rathi

Stress or temperature can change the lattice organization. Along with volume and density changes, there is a form shift. Superelasticity is the capacity to resist stress without enduring deformation and return to the original lattice structure. NiTi files have a shape-memory effect, which allows them to return to their native linear shape lacking signs of long-lasting distortion [8]. SMAs have an extremely reduced proof strength when the temperature is decreased or when the NiTi wire is under its transition temperature and may be readily molded in a new shape with a little loss of integrity. There is a switch in the structure of the crystal lattice of NiTi when it’s heated beyond its transition temperature, and this leads to the return of NiTi into its prior shape. NiTi wires used in orthodontics are referred to as nitinol, and these wires have two phases. At low temperature, they exist in martensitic also called as the daughter phase, and the lattice arrangement of the martensitic phase is in body-centered cubic (BCC) lattice. In contrast, at a temperature which is higher than normal, they acquire austenitic also called as the parent phase, and the lattice arrangement of the austenitic phase is a hexagonal lattice. Any kind of stress and temperature change can lead to an alteration in the lattice organization (Figure 1).

NiTi files undergo a stress-induced martensitic change from the austenitic to the martensitic phase during endodontic root canal therapy. Along with the change in volume and density, a form shift also takes place. NiTi files have a property of shape memory, which allows them to return to their original straight shape without displaying any signs of permanent deformation [9]. An SMA has an extremely low yield strength when cold, or below its transition temperature, and may be easily bent into any new shape with little loss of integrity. But when heated beyond its transition temperature, the file undergoes a change in crystal lattice structure that makes it return to its original form. During biomechanical preparation, the superelastic property of NiTi rotary instruments helps in getting a better access for canals that have curved paths. With a small amount of canal transit and a lower frequency of canal irregularities, it enables more concentrated canal preparations. Due to their reduced flexibility and tensile qualities, NiTi arch wires are preferred over stainless steel in orthodontics. Because of their superelasticity and shape memory, NiTi wires continuously exert moderate stresses on teeth for a longer period of time while they are within physiologic range [10].

Smart composites

**Figure 2:**
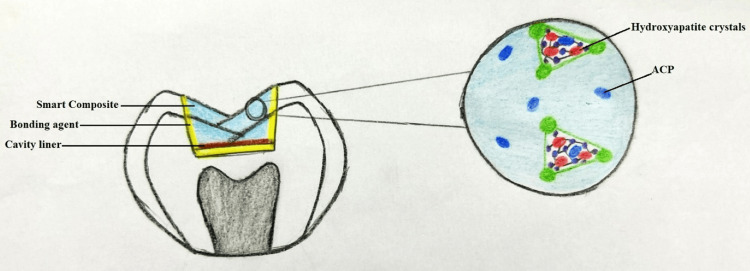
Formation of hydroxyapatite crystals ACP: amorphous calcium phosphate Image Credit: Harsha P. Rathi

Boskey stated that amorphous calcium phosphate (ACP) was first characterized by Aaron S. Posner in the middle of the 1960s [11]. ACP-containing products were created for a variety of uses such as liners, bases, pit and fissure sealants, etc. [12]. "ACP is a filler contained in a polymer binder that Skrtic has created specifically for biologically active restorative materials that can encourage the regeneration of hydroxyapatite crystals due to prolonged release of large amounts of Ca+ and Po+." The ACP-containing composites release Ca+ and Po+ into the saliva in addition to having good biocompatibility. As an apatitic material identical to the hydroxyapatite crystals (HAP) present naturally in the teeth and bones, these ions can be implanted inside the tooth structure (Figure 2) [13].

ACP is still the same at neutral or basic pH levels. ACP transforms into HAP and precipitates during a carious attack when the pH is low (at or below 5.8), replacing the HAP that was lost to the acid. Thus, these ions quickly combine to create a gel when the pH in the oral cavity is under 5.8. The gel transforms into amorphous crystals in two minutes or less, producing Ca+ and Po+ [14]. One could call the way ACP-containing composites react to pH smart. This smart behavior of the composite only leads to the remineralization of the tooth [15]. Disadvantages of the conventional composite such as poor wear resistance, bulk fracture in the area of stress, polymerization shrinkage, etc. are not paid attention to. These are the limitations for smart composites.

Self-healing composites

**Figure 3:**
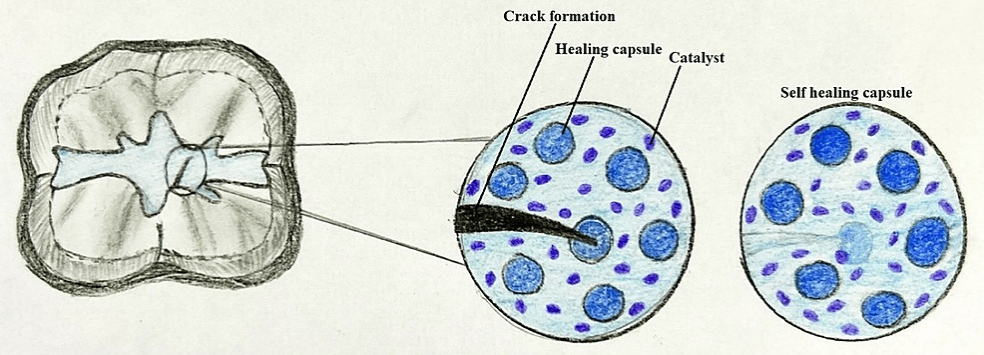
Mechanism of action of a self-healing composite Image Credit: Harsha P. Rathi

Scientists have created materials that can mend by itself inspired from nature. Various natural materials are actually "self-healing composite materials." Natural bone is a good illustration of this because it undergoes permanent remodeling and has the capacity of healing on its own even after suffering evident fracture. The making of bioinspired material systems is a major area of contemporary scientific study [16]. Most materials have a finite lifespan and deteriorate as a result of various physical, chemical, and/or biological stresses. This causes the structure of the material to gradually deteriorate and eventually fail [17]. Composites fixed using a self-repairing process formulated on microcapsules might be effective to a greater extent than those restored using macroscopic repair techniques, some of which have been found to not produce sufficient mechanical properties of the repaired composite with an appealing outcome [18]. A self-healing composite contains a healing powder and a healing liquid which is encapsulated by silica nanoparticles. The liquid contains water and polyacrylic acid. Silica particles increase the bond strength of the resin which also reduces the fracture of the material. If a crack develops in the self-healing composite, the silica particles enter into the crack and release the liquid which leads to a reaction between the liquid and powder, the end result of which is the formation of glass ionomer cement (GIC) which heals the crack. As GIC is formed, it also forms an ionic bond with the composite and immediately stops the progression of crack (Figure 3).

In a study, Wertzberger et al. looked at the physical characteristics of a model dental compound designed to automatically heal cracks as well as the effectiveness of self-healing in a heavily filled composite [19]. A model resin made of triethylene glycol dimethacrylate that cures under visible light is urethane dimethacrylate (UDMA). The self-healing system used in the formulation of bisphenol A-glycidyl methacrylate at 45% weight-for-weight with silane 0.7 glass included encapsulated dicyclopentadiene and Grubbs catalyst. The self-repairing material's fracture toughness was precisely comparable to the control.

Smart ceramics

The primary consideration for any dental treatment has always been and will continue to be aesthetics. Zirconia was created as a result of the widespread usage of porcelain fused to metal (PFM) restorations, which have various cosmetic drawbacks as a grey gingival discoloration. It is a polycrystalline ceramic that doesn't include glass and has a dense and regularly packed crystalline pattern that makes it harder to cause fracture propagation when we compare it with irregular and less dense glass ceramics. As a result, zirconia is far more durable. At the Swiss Federal Institute of Technology (ETH) Zurich, the first "all ceramic tooth bridge" was created in 1995 using a method that allowed direct machining of bridges without the need of stainless steel or metal. Since then, the procedure and the components have undergone testing before being released as smart ceramics. Bridges can be built, thanks to strength and technology. Zirconia is more fracture robust and has better flexural strength than any other ceramic material now on the market. Although the copings need to be veneered for greater aesthetics and are fairly opaque, they seem quite realistic. Due to its special characteristic of "transformation toughening," it is the ideal choice. In contrast to alumina, zirconia changes its crystalline structure upon burning. With 3-8% calcium and yttrium or cerium, the material has a tetragonal structure during fire and a monoclinic structure (4.4% higher volume) at room temperature to stabilize the tetragonal form (without which the material would shatter upon cooling) [20]. The crystals must undergo a highly confined stress prior to fracture propagation in order to change into their monoclinic form. The 4.4% volume increase caused a compressive fracture closure stress, which effectively slowed down or stopped the crack [21]. As a result, a compressive or crack closure stress is generated, which causes the fracture to slow down or stop. Zirconia is a smart material because of the crystallographic alteration it underwent in reaction to stress.

GIC (smart material)

Consumption of hot or cold food and liquids can cause significant thermal alterations in the mouth. As a result, the materials used for restoration in this environment might experience thermal expansion or contraction in reaction to thermal stimuli. Commonly used to characterize the dimensional changes a substance undergoes in response to heat change is the coefficient of thermal expansion (CTE) [22]. Gap development at the interface is almost nonexistent when two materials increase or decrease in size at comparable rates; as a result, microleakage is minimal [23,24].

When glass ionomers are heated and cooled at 20-50°C in moist environment, little to no change in size was seen. When heated over 50°C in dry circumstances, the materials displayed a noticeable contraction. This phenomenon may be explained by the fact that fluid flow to the surface of the material compensates for the anticipated expansion upon heating to balance the dimensional changes [25]. The procedure was reversed after cooling. The observed shrinkage in dry circumstances is caused by the quick loss of water upon heating. This behavior is comparable to that of human dentine, which exhibits a substantial contraction when heated in dry settings but minimal dimensional change when heated in moist conditions [26]. The fluid flow in the dentinal tubules can be used to explain both outcomes. As a result, it can be claimed that the glass ionomer materials exhibit a form of intelligent behavior that mimics the behavior of human dentine [27].

GICs lose mass in moist circumstances much lower when compared with dry circumstances. GICs may exchange fluid with the environment since they are made of water. Due to changes in the surrounding, loosely bonded fluid is easily lost and found again. The process of losing loosely bonded fluid is probably reversible; the water may be reabsorbed upon cooling. Because of their active response to their surroundings, GICs might be said to exhibit "smart" behavior. This "smart" behavior may have been "triggered" by the first water loss brought on by the environment's temperature shift. Porosity is a built-in characteristic in GICs [28,29]; due to this property, fluid gets accumulated in the porosities which might greatly increase the fluid content in GIC.

GIC is set to be a smart material in regard to its thermal characteristics, because it is a desirable attribute when restorative material undergoes temperature-induced volumetric changes equal to those of the tooth materials [30].

SmartSeal obturation system

Antimicrobial treatment of necrotic pulp tissue is difficult due to the microorganisms that are attached in the pulp space to the infected dentin [31]. Obturation of the canal is intended to stop periradicular illness by preventing reinfection of the root canal. Reinfection prevention can be done by filling the prepared root canal, accessory canal, and dead spaces with proper 3D seal. The C-point system is a point-and-paste method for filling root canals that uses endodontic points which are hydrophilic [32]. Trogamid T and Trogamid CX (Evonik Industries, Essen, North Rhine-Westphalia, Germany), two exclusive nylon polymers, are combined to form the inner core of the C-point. According to claims, the lateral expansion of C-point happens unevenly, and the expandability depends on how much the hydrophilic polymer has been prestressed [33].

This non-isotropic lateral expansion is thought to improve the capacity of the root canal filling to seal, lowering the risk of reinfection and enhancing the long-term effectiveness of root canal therapy. Voids might still exist in the walls of root canals and the extended point even if C-point is capable of attaining a rather excellent fit of an uneven canal space. In order to seal certain regions, an additional sealer must be employed [34].

By assessing the cell survival and mineralization potential of the rat odontoblast-like MDPC-23 cell line, Eid et al. assessed the biocompatibility of C-point and commercially available gutta-percha points [35]. After the elution of potentially hazardous components, they came to the conclusion that C-point's biocompatibility in artificial conditions is equivalent to gutta-percha with low negative result on the formation of bone [36].

Smart coatings for dental implants

**Figure 4:**
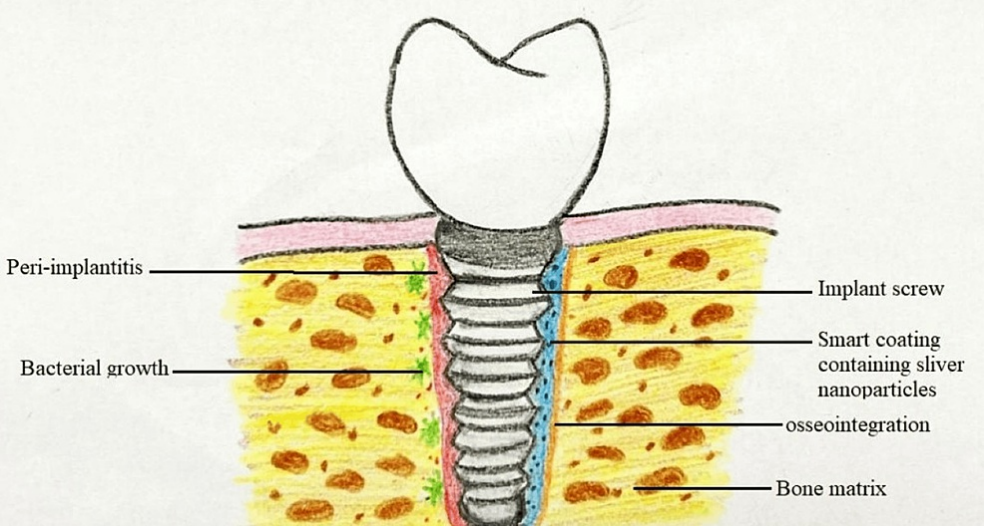
Smart coating showing osseointegration of dental implants Image Credit: Harsha P. Rathi

A "smart coating" created by scientists at North Carolina State University (NC State) enables surgical implants to adhere to bone more tightly and reduces the risk of infection [4]. This has made safer dental, knee, and hip implants possible. NC State-developed smart covering reduces the danger of implant rejection by encouraging the bone to grow in the implant. An exterior coating is formed which meets the surrounding bone that is primarily amorphous and forms a crystalline layer adjacent to the implant. As the amorphous layer disintegrates over time, calcium and phosphate are released, promoting bone formation. As the amorphous layer dissolves, the coating is absorbed by the bone, enhancing osseointegration (Figure 4). 

The bonding also improves the functionality of the implant by ensuring that the bone and the implant more effectively distribute the load. Due to the fact that everyone's rate of bone formation differs greatly, this is significant. For instance, the bones of young individuals often develop far more quickly than the bones of older folks [4].

To prevent infections, the researchers have added silver nanoparticles all over the coating [4]. Currently, implant patients are put through a rigorous antibiotic treatment immediately following surgery to avoid infection. The implant site, however, will always be prone to infection. But when the amorphous layer breaks down, the silver particles that were included into the coating will function as antibacterial agents. The rate of silver release will decrease as the patient heals [37]. The authors refer to it as "smart coating" for this additional reason [38]. Table 1 shows the unique properties and uses of all the smart materials mentioned above.

**Table 1:** Unique properties and uses of smart materials NiTi: nickel-titanium; ACP: amorphous calcium phosphate Table Credit: Harsha P. Rathi

Sr. no.	Smart material	Unique properties	Uses
1)	NiTi smart alloy	Shape memory. Phase transition. Superelasticity.	Biomechanical preparation of the root canals. NiTi arch wires in orthodontics.
2)	Smart composites	Contains ACP extended time release. Formation of hydroxyapatite crystals.	Class 1 and class 2 cavity in primary and permanent teeth up to 4mm depth.
3)	Self-healing composites	Defends against material failure and improves the safety and reliability of composite.	Class 1 and class 2 restorations (in patients with high occlusal forces).
4)	Smart ceramics	Transformation toughening.	Bridges. Crowns. Pontics.
5)	Glass ionomer cement (smart material)	Temperature-induced volumetric changes which are equal to that of dentin.	Liners. Bases. Reinforced restorative material.
6)	SmartSeal obturation system	C-point system which is hydrophilic in nature. Non-isotropic lateral expansion.	Obturating material.
7)	Smart coatings for dental implants	Enhancing osseointegration. Presence of silver nanoparticles which prevents infection.	Dental implant placement.

## Conclusions

Because of the lack of certain mechanical/chemical properties in conventional materials, the clinical outcome of the treatment may not last for a long time. Therefore, there is a need to increase the research in this arena to get the best outcome of the treatments. The application of smart biomaterials has led to a new revolution in dentistry. Active research is going on in this field to implement more such biomaterials that are beneficial to dentists for delivering high-quality treatment to patients. Different dental treatment methods will undergo a complete transformation as a result of the development of these newer, superior smart materials, making them more convenient for both the operator and the patient. A dentist's investment in these smart materials will undoubtedly be a wise one given their constantly increasing level of intelligence. Based on their capacity for recognition, analysis, and discrimination, these bioresponsive materials are able to foresee problems. There is always room for greater advancement, which will eventually result in achieving an excellent effect of dental treatment processes.
